# A slow-release strategy of *Lactobacillus plantarum* to enhance the degradation of cellulose by *Bacillus methylotrophic* in the ensiling process of corn stalk

**DOI:** 10.3389/fmicb.2024.1463645

**Published:** 2024-12-04

**Authors:** Yongqing Wan, Siyuan Liu, Yunhao Song, Ruihua Tian, Ruigang Wang, Kaihong Duan, Mandlaa Mandlaa

**Affiliations:** ^1^College of Life Science, Inner Mongolia Agricultural University, Hohhot, China; ^2^College of Food Science and Engineering, Inner Mongolia Agricultural University, Hohhot, China

**Keywords:** corn stalk, silage, slow-release, *Bacillus methylotrophicus*, *Lactobacillus plantarum*

## Abstract

The enhancement of cellulose degradation is important for improving the quality of corn-stalk silage. However, the rapid drop in pH caused by the propagation of lactic acid bacteria (LAB) can influence the degradation of cellulose by cellulose-degrading microorganisms (CDMs) during the mixed fermentation process of ensilage. In this study, a CDM (*Bacillus methylotrophic* 2–4, BM 2–4) was isolated, and its lyophilization condition was studied. Moreover, a slow-release strategy was developed to delay the release of LAB (*Lactobacillus plantarum* S-1, LP S-1) by embedding technology to provide time for BM 2–4 to degrade cellulose during the corn-stalk ensilage process. The results showed that BM 2–4 had a higher survival rate (89.53%) under the following conditions: cell collection (5,600 r/min in 4.4°C for 9.5-min centrifugation) and lyophilization using cryoprotectants [skim milk (10.4%), peptone (10.4%), and glucose (5.2%)] at −50°C with a vacuum pressure of <10 Pa. Based on the results of the previous study on embedded LP S-1, simultaneous inoculation of embedded LP S-1 and lyophilized BM2-4 at a 2:1 ratio, with an inoculum size of 6% and moisture content of 85%, significantly degraded *CF* by 3.8% and increased CP by 3.7% (*p* < 0.05). This treatment did not significantly influence the final pH of corn-stalk silage (*p* > 0.05) after 7 days of fermentation.

## 1 Introduction

Corn (*Zea mays* L.) is one of the major grains in the world, and it is estimated that 10 million tons of corn are produced annually (International Grains Council, IGC). Such a large amount of corn production has led to the problem of corn-stalk utilization (Hong et al., 2016; Cao et al., 2024). Among the utilization methods of corn stalks, silage is a cost-effective and environmentally friendly strategy for large-scale and long-term preservation of corn stalks (Tao et al., 2020). The anaerobic and acidity conditions formed in the ensilaging process can protect corn stalks from harmful bacteria and fungi (Yang et al., 2001) and prolong the storage time of corn stalks with minimal nutrient loss.

There are many ways to improve the quality of corn-stalk silage (Yang et al., 2001; Lara et al., 2016; Drouin et al., 2019), and the technique of enhancing the degradation of cellulose is the key to improving the palatability and preservation of corn-stalk ensilage for feed (Yang et al., 2001; Sun et al., 2018). It is reported that physical pretreatment (Cao et al., 2023; Zhao et al., 2018) and chemical pretreatment (Zhou et al., 2011) can significantly improve the degradation of cellulose before ensilage. These approaches can decompose or remove lignin and degrade hemicellulose by changing the macrostructure and crystallinity of cellulose to increase the contact area and improve the hydrolysis efficiency of cellulose (Niju and Swathika, 2019; Tinôco et al., 2021; Wang J et al., 2021). However, special equipment for physical pretreatment and toxic byproducts from chemical pretreatment had negative effects on the production process and quality of silage. Moreover, large-scale centralized pretreatment also increases transportation costs.

Although there are various cellulose-degradation microorganisms (CDMs) on the surface of raw material of silage (Li et al., 2021), they are inhibited by the decline of pH caused by the rapid propagation of lactic acid bacteria (LAB) in the ensilage process (Awasthi et al., 2018). To avoid the contradiction between pH and CDM in the ensilage process, a two-stage process was developed by Yang et al. (2001), and the ensilage process was divided into aerobic fermentation to degrade cellulose by CDM and anaerobic ensiling by LAB. The two-stage process can significantly improve the cellulose degradation of raw materials and enhance the quality of silage, but it inevitably increases the manipulation in the ensiling process.

Based on the above, a hypothesis was proposed that if the facultative anaerobic CDM was used together with slow-release LAB at the same time to ensilage the raw materials, it could achieve the goal of enhancing cellulose degradation and silage quality through a one-stage ensilaging process. To prove this hypothesis, LAB, *Lactobacillus plantarum* S-1 (LP S-1), was selected and embedded by an agent containing corn stalks to delay LP S-1 release in our previous study (Song et al., 2018). In this study, a facultative anaerobic CDM (*Bacillus methylotrophicus* 2–4, BM 2–4) was evaluated, and the conditions of lyophilization were studied. Moreover, the effects of inoculating embedded LP S-1 and lyophilized BM 2–4 on crude fiber (*CF*), crude protein (CP), and pH of corn-stalk silage were investigated.

## 2 Materials and methods

### 2.1 Microorganism

*Lactobacillus plantarum* S1 (LAB) was isolated from the corn stalks, and CDMs with higher CMCase activity sizes were isolated from rotted corn stalks (Song et al., 2017).

### 2.2 Corn stalk

The corn stalks (moisture content of 75%) were obtained from Hohhot, Inner Mongolia, China. After natural drying, the corn stalks were divided into two groups. Group 1: the corn stalks were pulverized and passed through a 40-mesh sieve to embed LP S-1 and culture CDM as the substrate. Group 2: the corn stalks were cut into 1–3 cm segments for the ensiling process.

### 2.3 Medium

Solid MRS medium: peptone 10 g (AR, Beyotime), beef extract 10 g (AR, Beyotime), yeast extract 5 g (AR, Beyotime), glucose 20 g (AR, TCI), diammonium hydrogen citrate 2 g (AR, Aladdin), Tween 80 1 mL (AR, Aladdin), CH_3_COONa 5 g (AR, Beyotime), K_2_HPO_4_ 2 g (AR, Beyotime), MgSO_4_ 0.58 g (AR, Solarbio), MnSO_4_ 0.25 g (AR, Solarbio), and agar 18 g (AR, Solarbio) were dissolved in 1 L distilled water and sterilized at 115°C for 35 min after pH was adjusted to 6.2–6.6. Solid cellulose medium: CMC-Na 5 g (AR, Beyotime), agar 20 g, K_2_HPO_4_ 1 g, MgSO_4_ 0.5 g, (NH_4_)_2_SO_4_ 2 g (AR, Beyotime), and NaCl 0.5 g (AR, Beyotime) were dissolved in 1 L distilled water and sterilized at 115°C for 35 min. Liquid corn-stalk medium: 10 g of corn stalks was added into 1 L distilled water and sterilized at 115°C for 35 min. Liquid cellulose medium: CMC-Na 10 g, K_2_HPO_4_ 1 g, MgSO_4_ 0.5 g, (NH_4_)_2_SO_4_ 2 g, NaCl 2.5 g, beef extract 2.5 g, and peptone 5 g were dissolved in 1 L of distilled water and sterilized at 115°C for 35 min. Liquid L. medium: Peptone 5 g, beef extract 5 g, yeast extract 5 g, and glucose 20 g were dissolved in 1 L distilled water and sterilized at 115°C for 35 min.

### 2.4 Evaluation of the cellulose-degradation ability of CDM

The isolates of CDM were inoculated into a liquid corn-stalk medium. After 15 days of fermentation at 30°C, the weightlessness and the concentration of soluble sugar were determined to evaluate the ability of degradation of corn-stalk cellulose.

### 2.5 Preparation of lyophilized CDM

The preparation of lyophilized CDM was divided into two steps: the collection conditions of CDM cells and the conditions of lyophilization of CDM. The single-factor experiment (SFE) and response surface methodology (RSM) were used to optimize the collection of cells and the selection of the best cryoprotectant in the preparation process. First, the effect of centrifugation, the time of centrifugation (0, 10, 20, 30, and 40 min), the temperature of centrifugation (−4, 0, 4, 8, and 10°C), and the rate of rotation (2,000, 4,000, 6,000, and 8,000 r/min) on the survival rate of BM 2–4 were evaluated using the SFE. Moreover, the best condition of cell collection was optimized using the RSM. Second, the effect of cryoprotectant, maltodextrin (5, 10, and 20%), skim milk (5, 10, and 20%), peptone (5, 10, and 20%), trehalose (5, 10, and 20%), and glucose (5, 10 and 20%) on the survival rate of BM 2–4 was evaluated using the SFE, and the composition of cryoprotectant was optimized using RMS. In addition, the process of lyophilization is as follows: the cells of CDM (adding cryoprotectant) were pre-frozen at −20°C after centrifugation and then transferred into a freeze dryer for lyophilizing (−50°C, vacuum pressure of <10 Pa). The number of microbial cells was counted using a hemacytometer, and the microbial survival rate was calculated to evaluate the conditions of lyophilizing CDM.

### 2.6 Preparation of the embedded LP S-1

The preparation process for embedding LP S-1 was described in our previous study. The embedded process consisted of two steps, and the brief process was as follows: LP S-1 was adsorbed with corn-stalk powder, followed by the addition of sodium alginate (1.2%) and CMC-Na (0.5%) to 4% CaCl_2_ for 24 h (Song et al., 2018).

### 2.7 The effect of inoculating lyophilized BM2-4 on the stability of embedded LP S-1

Lyophilized BM2-4 and embedded LP S-1 were inoculated into a liquid corn-stalk medium (static cultivation at 30°C) to evaluate the effect of lyophilized BM2-4 on the stability of embedded LP S-1 by recording the residual number of embedded balls of LP S-1 during the fermentation process.

### 2.8 The effect of inoculating embedded LP S-1 and lyophilized BM2-4 on the concentration of *CF* and CP in corn-stalk silage

SFE was used to investigate the effect of inoculum size (2, 3, 4, 5, 6, 7, and 8%), fermentation time (0.25, 0.5, 1, 2, 3, 5, 7, and 9 days), moisture content (45, 55, 65, 75, 85, and 95%), and proportion (embedded LP S-1:lyophilized BM2-4, 4:1, 3:1, 2:1, 1:1, 1:2, 1:3, and 1:4, g/g) on the concentration of crude fiber (*CF*), crude protein (CP), and pH in the ensiling process of corn stalks. The mixed fermentation broth (LP S-1 and BM2-4) and embedded LP S-1 were used as a control to evaluate the performance of this slow-release strategy. The experiment was conducted in the anaerobic fermentation bags (50 × 30 cm) at 30°C; CP and *CF* were determined at the beginning and end of the ensiling process.

### 2.9 Assay methods

The concentration of soluble sugar was determined using the DNS method (Sun et al., 2006), and the survival rate of lyophilization was determined using the method of Shao et al. (2014). The determination of CP and *CF* was carried out by the description in Chinese National Standards of GB/T 6432–94 and GB/T 6434–2006. pH was determined using a pH meter.

The rate of weightlessness is calculated by Equation 1.


1 $$\mathrm{The rate of weightlessness}=\frac{W-w}{W}\times 100\%$$

w: The weight of corn stalk after ensiling.

W: The weight of corn stalk before ensiling.

The number of living microbial cells was counted by the method of hemacytometry, and the microbial survival rate was calculated using Equation 2.


2 $$\mathrm{The rate of microbial servival}=\frac{m}{M}\times 100\%$$

m: The number of living microbial cells after treatment per volume.

M: The number of living microbial cells before treatment per volume.

The rate of cellulose degradation is calculated using Equation 3.


3 $$\mathrm{The rate of cellulose degradation}=\frac{C-c}{C}\times 100\%$$

c: The concentration of crude fiber after ensiling.

C: The concentration of crude fiber before ensiling.

The rate of protein increase is calculated using Equation 4.


4 $$\mathrm{The rate of protein increase}=\frac{P-p}{p}\times 100\%$$

p: The concentration of crude protein before ensiling.

P: The concentration of crude protein after ensiling.

### 2.10 Statistical analysis and plotting

Three replications were performed in all treatments, and a one-way analysis of variance (ANOVA) was used to evaluate the differences between the two groups. The difference between the two groups was regarded as statistically significant if *p* < 0.05. Design Expert 8.05b was used to design and analyze the RSM. Figures were drawn by Microsoft Office Excel 2023.

## 3 Results and discussion

### 3.1 Selection of CDM

**Figure 1:**
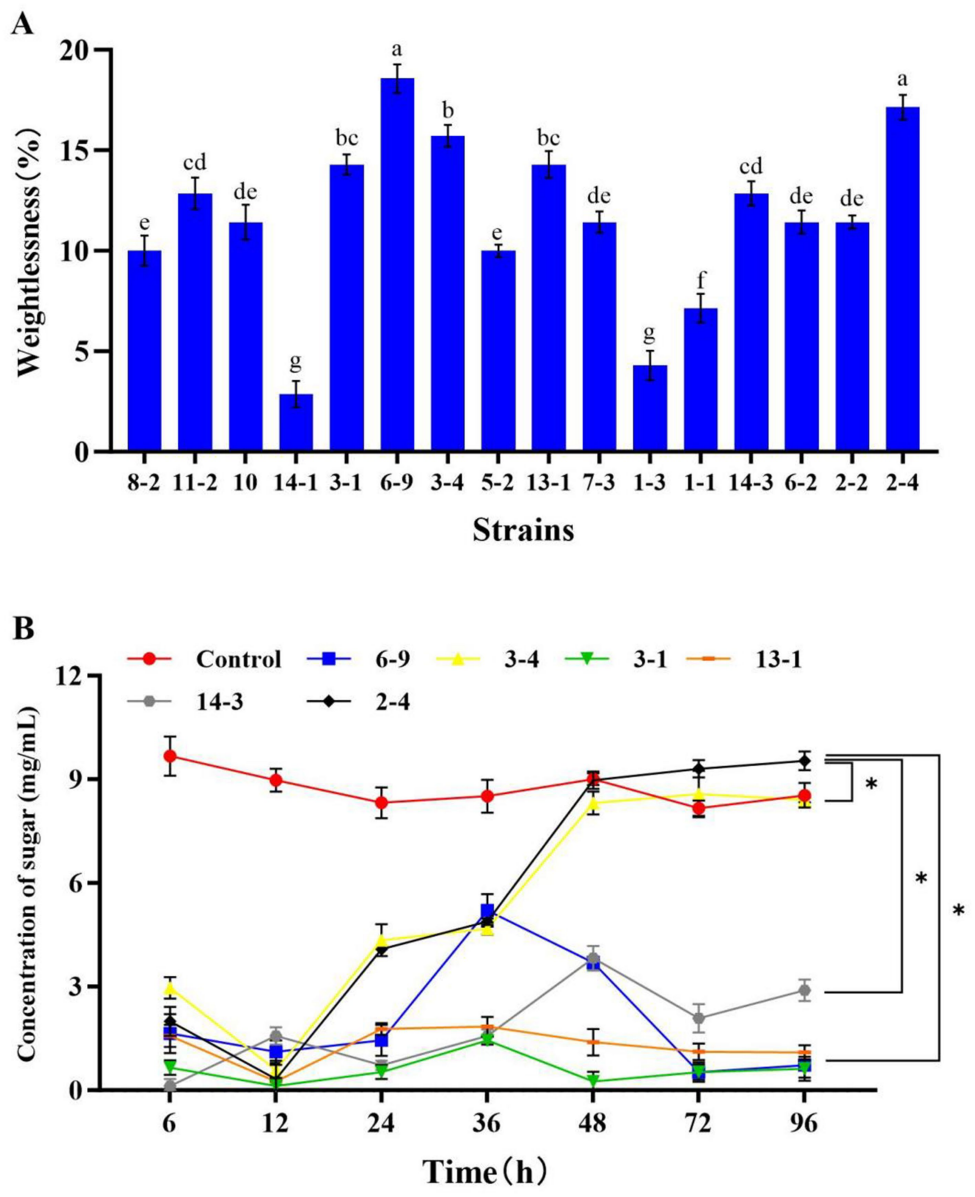
Effect of the isolates on the rate of weightlessness and concentration of soluble sugar in the fermentation broth of corn stalk. The experiment was repeated three times, and the error lines represent the SD; values with different small letters indicate significant differences among treatments; *p < 0.05. **(A)** Rate of weightlessness after 15 days of fermentation of 16 isolates. **(B)** Concentration of soluble sugar after 96 h of fermentation of 6 isolates.

In the previous study, 54 CDMs were isolated from rotted corn stalks, and 16 of them with higher CMCase activity were identified (the majority belonging to the genus *Bacillus*; Song et al., 2017). In this study, the corn-stalk powder was used as the substrate to select the CDM with a higher ability of corn-stalk degradation from the 16 isolates. The results showed that six isolates (6–9, 3–4, 3–1, 14–1, 2–4, and 14–3) had higher weightlessness (Figure 1A), and the weightlessness of them was significantly higher than others (*p* < 0.05). This indicated that these isolates had a higher ability of corn-stalk degradation. Moreover, a contrast experiment of the six isolates was conducted, and the soluble sugar was determined to further evaluate the ability of cellulose degradation. The results showed that five isolates (3–1, 6–9, 3–4, 14–1, and 2–4) consumed the pre-existing soluble sugar in 0–12 h and began to produce sugar after 12 h of fermentation. The concentration of soluble sugar in the fermentation broth of 3–4 and 2–4 reached a plateau at 48 h, and the concentration of soluble sugar in the fermentation broth of 2–4 (9.54 mg/mL) was significantly higher than other isolates after 96 h of fermentation (*p* < 0.05, Figure 1B). Therefore, 2–4 was selected as CDM for further study in this study. Many microorganisms can produce cellulase (Lynd et al., 2002). It was reported that the genus *Bacillus* not only can degrade cellulose (Duman et al., 2016; Saffari et al., 2017; Yadav et al., 2024) but can also inhibit yeasts, molds, and certain harmful microorganisms (Lara et al., 2016; Li et al., 2024), thereby improving the quality of feed and performance of livestock growth (Zhang et al., 2016). *B. methylotrophicus* has the effect of inhibiting fungal growth (Li et al., 2016; Tumbarski et al., 2018; Jiang et al., 2019) and it may have positive effects on the corn-stalk silage. In this study, 2–4 was identified as *Bacillus methylotrophicus* (BM 2–4, a facultative anaerobe) in our previous study (Song et al., 2017).

### 3.2 Preparation of lyophilized BM 2–4

**Figure 2:**
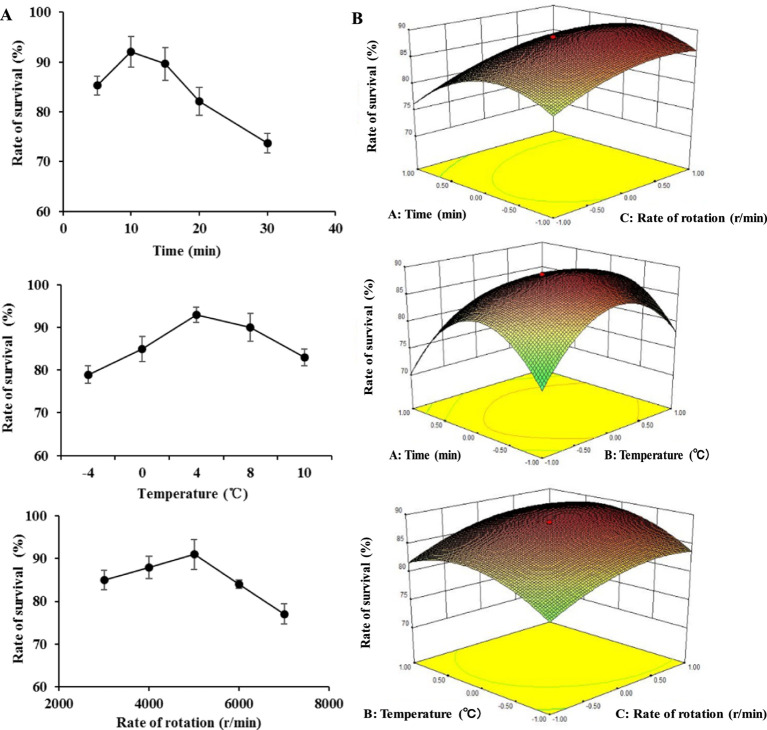
Effect of collecting conditions on the survival rate of BM 2–4. The experiment was repeated three times, and the error lines represent the SD. **(A)** Effect of time, temperature, and rotation rate of centrifugation on the survival rate of BM 2–4. **(B)** Comprehensive effect of time, temperature, and rotation rate of centrifugation on the survival rate of BM 2–4.

The whole lyophilization process of BM 2–4 was separated into two stages to obtain the maximum survival rate, cell collection, and lyophilization. In the first stage, parameters of cell collection were evaluated (SFE) and optimized (RSM) for collecting the cells of BM 2–4. After SFE, the optimal rates of rotation, centrifugation time, and temperature of centrifugation were 5,000 rpm/min, 10 min, and 4°C, respectively (Figure 2A), and the results of RMS showed that the maximum survival rate was 89.21% at the conditions of 5,600 r/min in 4.4°C for 9.5-min centrifugation (Figure 2B).

**Figure 3:**
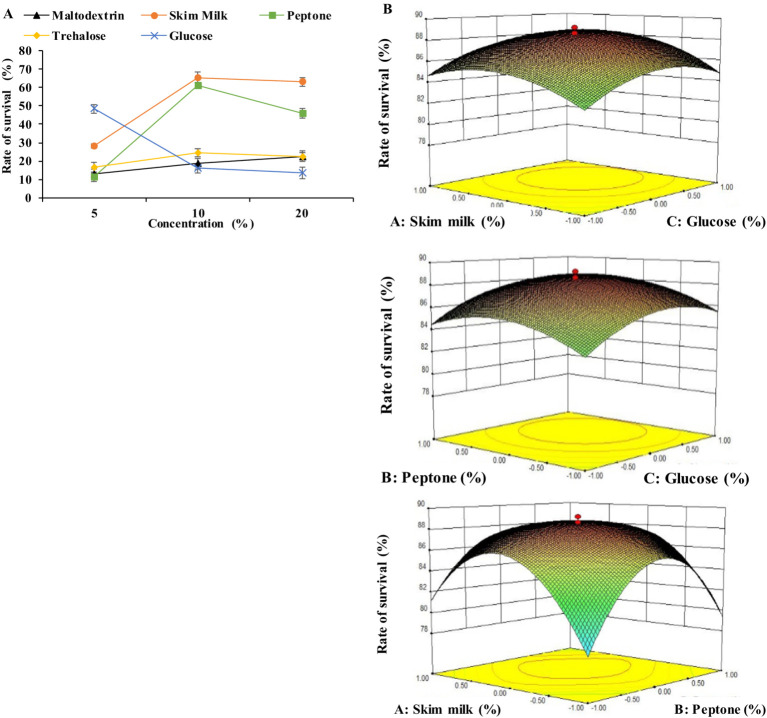
Effect of lyoprotectants on the survival rate of BM 2–4. The experiment was repeated three times, and the error lines represent the SD. **(A)** Effect of the concentration of lyoprotectants on the survival rate of BM 2–4. **(B)** Comprehensive effect of the concentration of lyoprotectants on the survival rate of BM 2–4.

In the second stage, the effect of five cryoprotectants (maltodextrin, skim milk, peptone, trehalose, and glucose) on the survival rate of BM 2–4 in the process of lyophilization was investigated. The results showed that skin milk, peptone, and glucose had a positive impact on the survival rate of BM 2–4, with the optimal concentrations being 10.0, 10.0, and 5.0%, respectively (Figure 3A). Furthermore, the predicted highest survival rate of 88.85% was achieved under the conditions of 10.36% skim milk, 10.38% peptone, and 5.64% glucose as verified by RSM (Figure 3B). The concentration of skim milk, peptone, and glucose was adjusted to 10.4, 10.4, and 5.2%, respectively, and the survival rate of 89.53% was achieved using a verification test. Lyophilization is an effective method for preserving the vitality of microorganisms; however, the formation of ice crystals can damage the structure of the cell membrane, leading to decreased vitality and metabolic activity (Beney and Gervais, 2001; Araújo et al., 2020). Cryoprotectants have a positive effect on the microbial survival rate in the lyophilization process and they can protect cells from dehydration (Nunes et al., 2018; Cheng et al., 2022). Skim milk can protect the cells from intense environmental change, and it has been used more widely than other cryoprotectants. Fonte et al. (2015) and Peiren et al. (2015) proved that different kinds of cryoprotectants work together to obtain a higher survival rate after lyophilization. In this study, skim milk, peptone, and glucose were selected as lyoprotectants to improve the survival rate of BM 2–4 in the lyophilization process.

### 3.3 Influence of BM2-4 on the stability of embedded LP S-1

**Figure 4:**
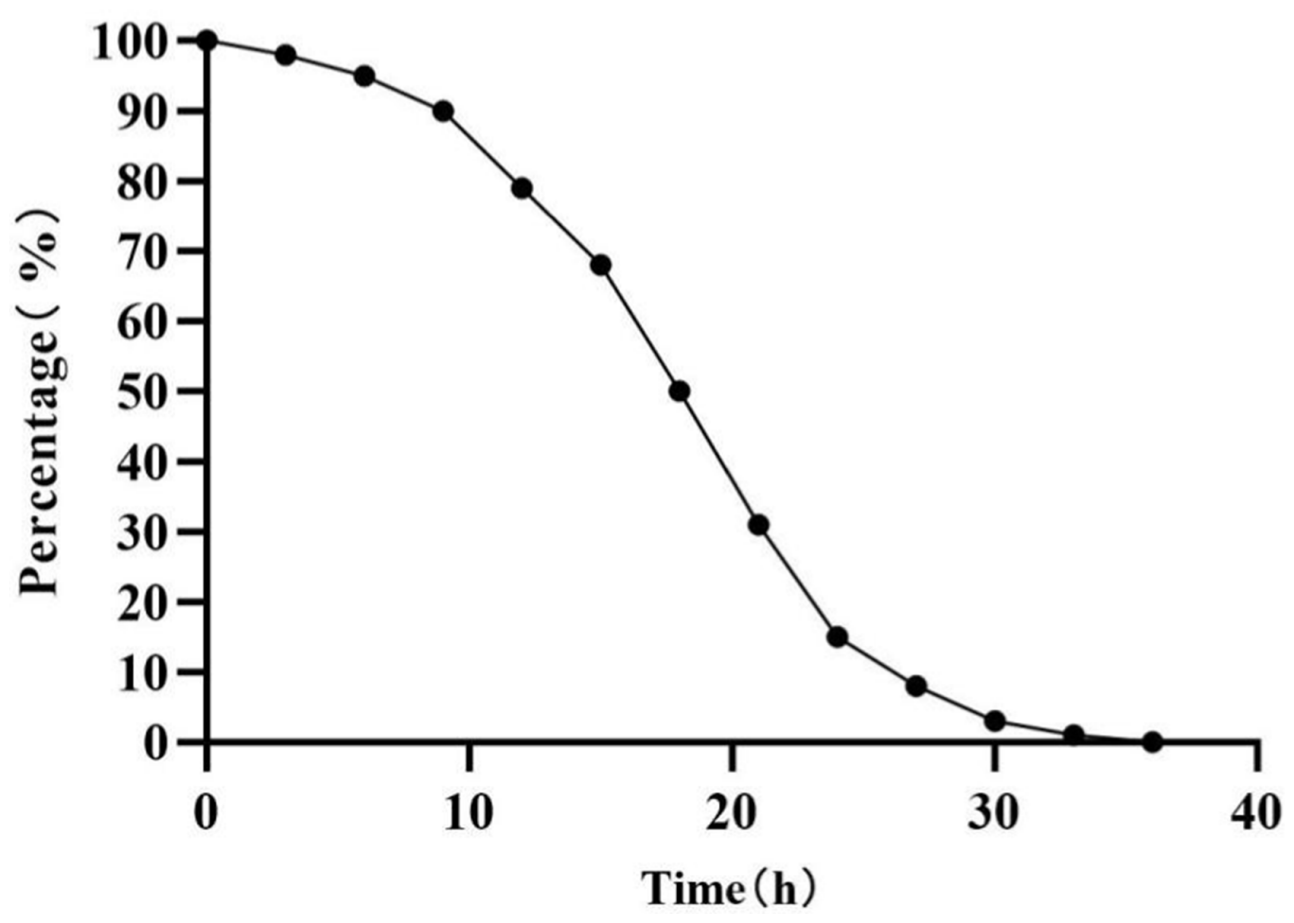
Influence of the BM 2–4 on the stability of embedded LP S-1.

The stability of embedded LP S-1 in the fermentation broth of BM 2–4 was investigated, and the results are shown in Figure 4. After 36 h of fermentation, the number of embedded LP S-1 was decreased from 100 to 0 in static cultivation at 30°C. This indicated that BM 2–4 could release LP S-1 slowly by disrupting the structure of the embedding material. The degradation time of embedded LP S-1 was a key factor in the degradation of corn stalks by BM 2–4. In this study, LP S-1 was embedded in a mixture of materials (corn stalk powder and calcium alginate gel; Song et al., 2018). This setup laid the foundation for destroying the embedded material of LP S-1 by BM 2–4, leading to the production of soluble sugars that stimulate the growth of LP S-1 during the ensiling process. The calcium alginate gel is the most common material to immobilize the enzyme or microorganism. It is reported that the stability of calcium alginate gel immobilization was degraded at the low pH (Simó et al., 2017), and the feature can be advantageous when used as slow-release material in our study. In the medical field, the encapsulation of certain active substances in alginate substrates can protect drugs, prevent premature drug inactivation, delay the release of drugs, and enable drugs to reach the target point at a fixed time to complete targeted therapy. Moreover, calcium alginate gel has been proven to be a safe additive for food (Prevost and Divies, 1992) and is feasible for use in animal feed production.

### 3.4 The effect of inoculum size, fermentation time, moisture, and proportion of embedded LP S-1 and lyophilized BM2-4 on the *CF* and CP in the ensiling process of corn stalks

**Figure 5:**
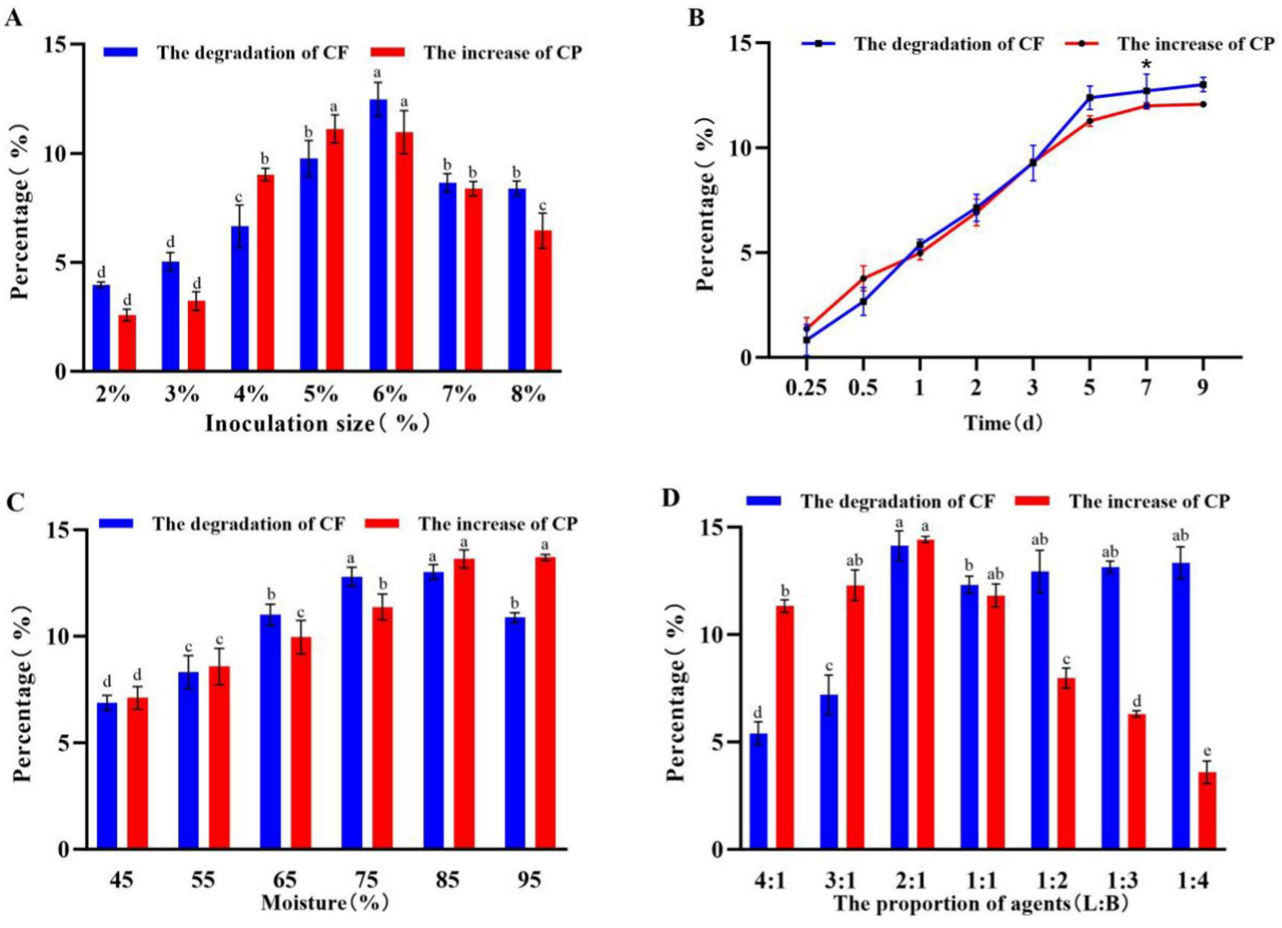
Effect of inoculation size, fermentation time, moisture, and proportion of slow-release ball S-1 and lyophilized 2–4 on the *CF* and CP of corn stalks. The experiment was repeated three times, and the error lines represent the SD; values with different small letters indicate significant differences among treatments; *p < 0.05. **(A)** Effects of the inoculation size on the *CF* and CP of corn stalks (2 days of fermentation, 55% moisture content, and LP S-1:BM 2–4 = 1:1). **(B)** Effects of the fermentation time on the *CF* and CP of corn stalks (6% inoculation size, 7 days of fermentation, and LP S-1:BM2-4 = 1:1). **(C)** Effects of the moisture on the *CF* and CP of corn stalks (6% inoculation size, 55% moisture content, and LP S-1:BM 2–4 = 1:1). **(D)** Effects of the cell number proportion of agent (LP S-1:BM 2–4) on the *CF* and CP of corn stalks (6% inoculation size, 85% moisture content, and 7 days of fermentation).

The effects of inoculum size, proportion of embedded LP S-1 and lyophilized BM2-4, fermentation time, and moisture on the *CF* and CP of corn-stalk silage were investigated, and the results are shown in Figure 5. Higher degradation of *CF* and increased CP (*p* < 0.05) in the ensiling process of corn stalks were obtained under the following conditions: 6% inoculum size (Figure 5A), 7 days of fermentation (Figure 5B), 85% moisture content (Figure 5C), and a 2:1 ratio of embedded LP S-1 and lyophilized BM2-4 (Figure 5D), respectively.

### 3.5 The effect of inoculating embedded LP S-1 and lyophilized BM 2–4 on *CF*, CP, and pH in the ensiling process of corn stalks

**Figure 6:**
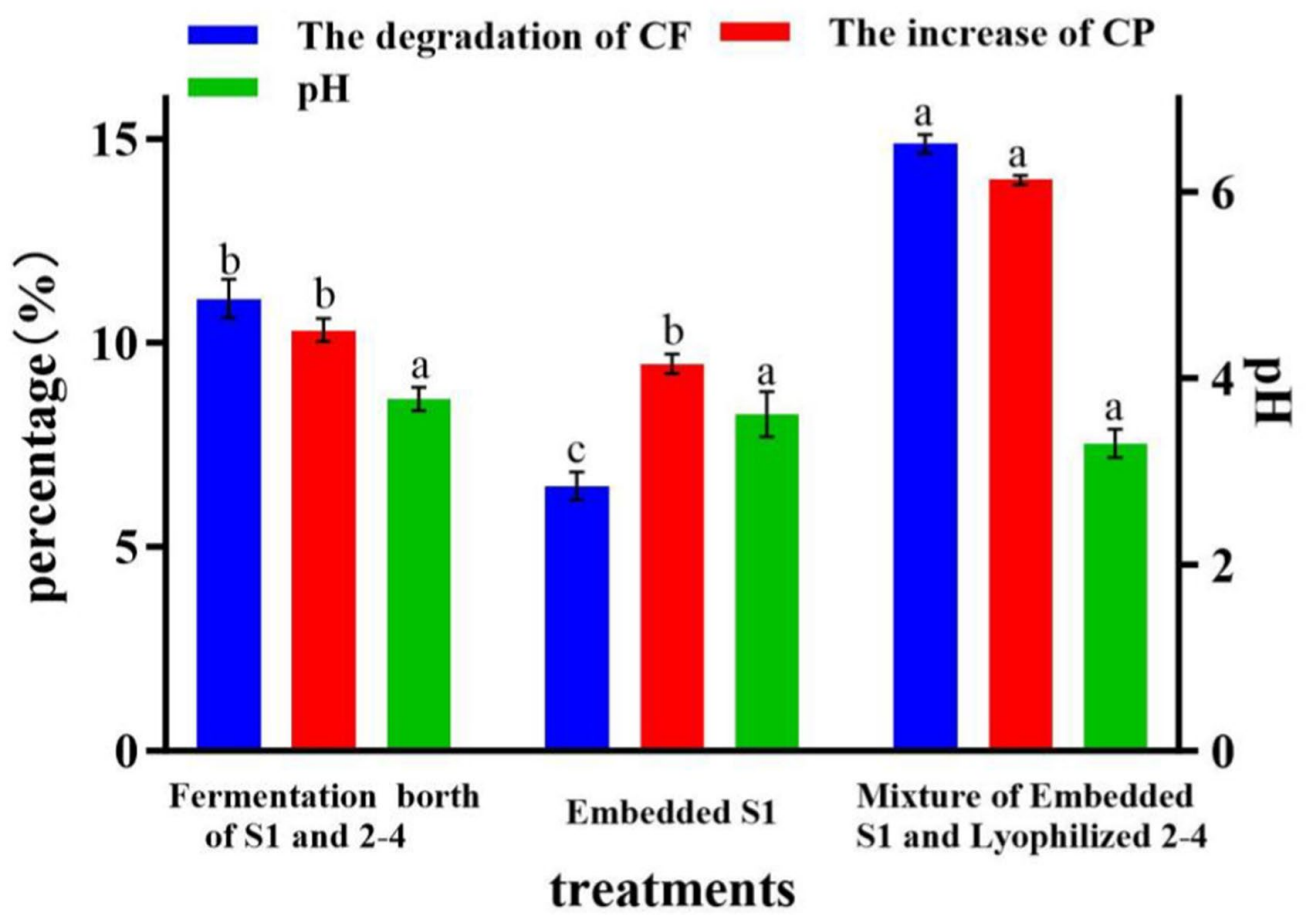
Effect of inoculating embedded LP S-1 and lyophilized BM 2–4 on *CF*, CP, and pH; values with different small letters indicate significant differences among treatments. The experiment was repeated three times, and the error lines represent the SD.

Based on the results of Section 3.4, the mixed fermentation broth (LP S-1 and BM 2–4) and embedded LP S-1 were used as the control (the concentration of cells is the same) to evaluate the effect of inoculating embedded LP S-1 and lyophilized BM 2–4 on *CF*, CP, and pH at the end of the ensiling process of corn stalk. After 7 days of fermentation, inoculating embedded LP S-1 and lyophilized BM 2–4 can significantly degrade *CF* by 3.8% and increase CP by 3.7% compared to inoculating the mixed fermentation broth of LP S-1 and BM 2–4 (*p* < 0.05, Figure 6). In addition, inoculating embedded LP S-1 and lyophilized BM 2–4 had no significant effect on the final pH compared to the mixed fermentation broth of LP S-1 and BM 2–4 (*p* > 0.05). This indicated that inoculating embedded LP S-1 and lyophilized BM 2–4 has a positive effect on the silage.

In the ensilaging process, LAB can secrete lactic acid and improve the flavor of silage (Liu et al., 2016). However, lactic acid can inhibit the growth and activity of other microorganisms including CDM (Wang Y, et al., 2021). The decline of pH caused by LAB was the main factor inhibiting the growth of CDM and the activity of cellulase. Therefore, the separation of LP S-1 and BM 2–4 to work at different times (BM2-4 first) can improve the degradation of cellulose in the ensiling process of corn stalk. Compared to the two-step process and pretreatment of corn stalks (Yang et al., 2001; Krafft et al., 2020) for silage, the slow-release strategy also can degrade cellulose and pH in the ensilaging process through a one-step operation.

## 4 Conclusion

The pH decrease caused by LAB was the main factor for reduced cellulose degradation during the mixed microorganism (LAB and CDM) ensiling process. In this study, a slow-release strategy of LAB was developed by embedding technology to verify the hypothesis that slow-release LAB can enhance the cellulose degradation of CDM without the influence of final pH in the corn-stalk ensilaging process. The results showed that the inoculation of embedded LP S-1 and lyophilized BM 2–4 with a 2:1 ratio, inoculum size of 6%, and moisture content of 85% can degrade *CF* by 3.8% and increase CP by 3.7% after 7 days of fermentation. Moreover, inoculation of mixed agents could not significantly influence the final pH of corn-stalk silage.

## Data Availability

The original contributions presented in the study are included in the article/supplementary material, further inquiries can be directed to the corresponding author.
